# Supplementary material for: Willingness of caregivers to donate a kidney to a patient with end-stage renal disease: Findings from four dialysis providing health facilities in Uganda
Source: PLOS Glob Public Health. 2022 Apr 22;2(4):e0000287. doi: 10.1371/journal.pgph.0000287 (PMC10021730; doi:10.1371/journal.pgph.0000287)
Supplement: Attachment — Submitted filename: Response to Reviewers.docx [file pgph.0000287.s002.docx]

# Editorial comments

Your manuscript is missing the following sections: Introduction. Please ensure these are present, and in the correct order, and that any references to subheadings in your main text are correct. An outline of the required sections can be consulted in our submission guidelines here: <https://journals.plos.org/globalpublichealth/s/submission-guidelines#loc-parts-of-a-submission>. Please review your reference list to ensure that it is complete and correct. If you have cited papers that have been retracted, please include the rationale for doing so in the manuscript text, or remove these references and replace them with relevant current references. Any changes to the reference list should be mentioned in the rebuttal letter that accompanies your revised manuscript. If you need to cite a retracted article, indicate the article’s retracted status in the References list and also include a citation and full reference for the retraction notice.

**Response: Thank you for this comment. We have included the introduction in the revised manuscript.**

# Reviewers' comments:

# Reviewer #1

1. The title is good and it reflects on the context of the paper. The background information provided is adequate. The methodology is good as well but however, no information was provided on how the participants were selected across the four health facilities used in the study. Sample size was given but no adequate information on sample size computation, such as the formula used to calculate the sample size was not included. The discussion and conclusion are good but more details on the barriers to kidney donation by health caregiver can be added.

**Response: Thank you for these comments. Concerning the selection of participants across the four health facilities, we had earlier indicated that “We consecutively enrolled consenting caregivers aged ≥18 years at the respective study sites during the study period.” In the revised manuscript, we have added a new sentence that reads: “The sampling or selection of caregivers was proportionate to the size of the number of dialysis admissions during the study period at the respective health facilities.”**

**Regarding the sample size, we have made slight revision and have shown all the required computations as follows: “Sample size estimation was based on the one-sample proportion approach for an unknown outcome. There were 160 participants across the study sites as the population size for the finite population correction factor. We assumed a hypothesized percentage of outcome in the population as 50% with a margin of error of ±5% at 95% confidence level. We determined that 114 participants are needed based on Kish and Leslie formula. When we adjusted the estimated sample size for finite population correction of proportions and an additional 10% non-response rate to the estimated sample size of 114, the overall sample size required was 125 participants. The computation was as follows: no = Z^2^p(1-p)/d^2^, where no=unadjusted sample size, Z = Z-score at 95% confidence level, p = proportion of the outcome in the population and d = acceptable margin of error. Therefore, no = 1.96^2^ X 0.5(1-0.5)/0.05^2^ = 384.**

**Adjusting for finite population correction of proportions using the formula, n = no/[1+(no-1/N)], where n = adjusted sample size, no = 384 and N=160 (the finite population), n = 384/[1+(384-1/160)] = 114. With 10% non-response rate (11 participants), the overall sample size equals 125.”**

**Concerning the details on the barriers to kidney donation by caregivers, we have added the following sentence: “The barriers to willingness to donate a kidney to a patient with ESRD includes health systems concerns like the lack of confidence in healthcare provided to patients and fear that the surgery might be less successful; family concerns like negative influences from members; religious concerns especially the none acceptance of organ donation; and, personal factors such as loss of body image, death, and prolonged hospitalization that potentially leads to loss of employment.”**

# Reviewer #2

1. The chosen theme stands out for addressing a pathology of high worldwide prevalence, end-stage renal failure, in which the therapeutic possibilities are complex and the available treatment in which there is a gain in quality of life is kidney transplantation. The need for kidney donors is growing and still faces numerous barriers and the manuscript is right in bringing the facilitators and difficulties that must be addressed in the community to increase adherence to donations. The research is unprecedented in Uganda according to its authors and brings data from several countries on the subject, enriching the discussion, bringing elements of a broader personal, family and social nature, including legal issues.

**Response: Thank you.**

2. The study adhered to the Strengthening the Reporting of Observational Studies in Epidemiology (STROBE) guideline and uses a questionnaire that was previously improved, with the aim of qualifying its internal consistency, both in the language approach and its psychometric property.

**Response: Thank you.**

3. An element to be considered is the economy and culture of caregivers in Uganda, if it is done only by family and friends or if there are other population groups that are involved in this type of action, such as health workers, if they are paid or not, allowing better comparison with other countries.

**Response: Thank you for this comment. We have added a new sentence to highlight the issue of economy and culture of caregivers in Uganda concerning kidney or organ donation in general. The new sentence in the discussion section reads as follows: “In Uganda, kidney donation is only acceptable from matching family members. It is not clear whether the patients pay the donors as no formal regulations or legislation are in place. Family members may donate organs because it is culturally acceptable or tolerable compared to donations from other relatives and friends.”**

4. The epidemiological analysis is thorough, but the methods do not clarify whether the sample was selected randomly or intentionally and how the sample was selected.

**Response: Thank you for this comment. This issue was raised by reviewer #1 in comment #1 above.**

**Earlier, we stated that “We consecutively enrolled consenting caregivers aged ≥18 years at the respective study sites during the study period.” In the revised manuscript, we have added a new sentence that reads: “The sampling of caregivers was proportionate to the size of the number of dialysis admissions during the study period at the respective health facilities.”**
